# Population growth of two limno-terrestrial Antarctic microinvertebrates in different aqueous soil media

**DOI:** 10.1007/s11356-024-32905-x

**Published:** 2024-04-27

**Authors:** Jordan S. McCarthy, Kathryn E. Brown, Catherine K. King, Uffe N. Nielsen, Katie Plaisted, Stephanie M. N. Wallace, Suzie M. Reichman

**Affiliations:** 1 Centre for Anthropogenic Pollution Impact and Management (CAPIM), University of Melbourne, Parkville, VIC 3010 Australia; 2 School of BioSciences, University of Melbourne, Parkville, VIC 3010 Australia; 3 Environmental Stewardship Program, Australian Antarctic Division, 203 Channel Highway, Kingston, TAS 7050 Australia; 4 Hawkesbury Institute for the Environment, Western Sydney University, Locked Bag 1797, Penrith, NSW 2750 Australia

**Keywords:** Antarctica, Rotifer, Tardigrade, Culture, Soil elutriate, Limno-terrestrial

## Abstract

Terrestrial microinvertebrates provide important carbon and nutrient cycling roles in soil environments, particularly in Antarctica where larger macroinvertebrates are absent. The environmental preferences and ecology of rotifers and tardigrades in terrestrial environments, including in Antarctica, are not as well understood as their temperate aquatic counterparts. Developing laboratory cultures is critical to provide adequate numbers of individuals for controlled laboratory experimentation. In this study, we explore aspects of optimising laboratory culturing for two terrestrially sourced Antarctic microinvertebrates, a rotifer (*Habrotrocha* sp*.*) and a tardigrade (*Acutuncus antarcticus*). We tested a soil elutriate and a balanced salt solution (BSS) to determine their suitability as culturing media. Substantial population growth of rotifers and tardigrades was observed in both media, with mean rotifer population size increasing from 5 to 448 ± 95 (soil elutriate) and 274 ± 78 (BSS) individuals over 60 days and mean tardigrade population size increasing from 5 to 187 ± 65 (soil elutriate) and 138 ± 37 (BSS) over 160 days. We also tested for optimal dilution of soil elutriate in rotifer cultures, with 20–80% dilutions producing the largest population growth with the least variation in the 40% dilution after 36 days. Culturing methods developed in this study are recommended for use with Antarctica microinvertebrates and may be suitable for similar limno-terrestrial microinvertebrates from other regions.

**Supplementary Information:**

The online version contains supplementary material available at 10.1007/s11356-024-32905-x.

## Introduction

Extreme conditions in Antarctica mean that organisms living in these climates require adaptations to survive ephemeral water availability and subzero temperatures for much of the year. The terrestrial biota of Antarctica is limited compared to temperate regions; autotrophs are predominantly bryophytes (mosses and lichens) as well as terrestrial algae and cyanobacteria with the few endemic vascular species being limited to the Antarctic Peninsula (Cary et al. 2010). Macroinvertebrates such as annelids (e.g. earthworms) and arthropods (e.g. millipedes, isopods and spiders), that are characteristic of soil fauna in temperate and tropical regions, are absent in Antarctica. Instead, Antarctic terrestrial soil fauna consists of microinvertebrates including springtails, mites, nematodes, rotifers and tardigrades (Hogg et al. 2006; Nielsen and Wall 2013). Knowledge of the fauna of Antarctic soil ecosystems has historically been limited with most studies assessing invertebrate presence and abundance under field conditions (Freckman et al. 1997; Sinclair and Sjursen 2001; Adams et al. 2014; Fontaneto et al. 2015; Nielsen and King 2015; Baird et al. 2019; Ball et al. 2023). Recent advancements towards understanding their biology have been made with some taxa, including investigations of nematode life histories (De Tomasel et al. 2013; Wharton and Raymond 2015), gut microbiomes (McQueen et al. 2022; Parr McQueen et al. 2023) and studies of microinvertebrate genetics (Thorne et al. 2014; Xue et al. 2024).

Laboratory cultures provide reliable and consistent populations of organisms for laboratory-based research such as life history studies, environmental toxicology and other environmental stress studies (Altiero et al. 2015; Tsujimoto et al. 2019; Stone et al. 2021). Wild-sourced organisms tend to be more variable in age and condition which can increase variability in responses observed in experiments, relative to laboratory-cultured organisms. With the difficulty in accessing Antarctica and sensitivity of Antarctic environments to disturbance (Brooks et al. 2019), it is not always possible, nor desirable, to source new test organisms directly from the field. Thus, laboratory cultures extend our ability to study and increase our knowledge of Antarctic microinvertebrates and their sensitivity to environmental stressors.

Rotifers are found in both aquatic and terrestrial environments; in water, they are mostly planktonic (free-swimming) while those in terrestrial environments exist in interstitial water in soils, or moist vegetation like mosses or terrestrial algae (known as limno-terrestrial) (Ricci 1983; Wallace 1987; Ricci and Melone 2000). Bdelloid rotifers reproduce asexually and use their eponymous rotor-like mouth pieces to filter particulate food (including bacteria, algae and fungi) out of their aquatic environment (Ricci 2017). Tardigrades are similarly found in moist environments ranging from the terrestrial (e.g. moss and lichen, soil or leaf litter) to aquatic, including sediments (both marine and freshwater) and algal mats (Altiero and Rebecchi 2001; Glime 2017). Diets of tardigrades vary considerably by species and environment but commonly consist of plant cells, algae and bacteria, with fewer species being fungivores or carnivorous (Altiero and Rebecchi 2001; Bertolani 2001). Tardigrades have a range of reproductive modes and depending on the species can be asexual or sexual, hermaphroditic or dioecious (Sugiura and Matsumoto 2021).

A number of studies on the culturing of endemic Antarctic microinvertebrates have been published, including on nematodes (Adhikari et al. 2010; De Tomasel et al. 2013; Brown et al. 2020), rotifers (Dougherty 1964a; Dartnall 1992), tardigrades (Dougherty 1964b; Altiero et al. 2015) and springtails (McGaughran et al. 2010). However, with the exception of artificial pore water utilised by Brown et al. (2020), none of these studies have trialled analogues of the interstitial water in soil. Presently, the most common protocols used for culturing both tardigrades and rotifers utilise simple artificial freshwater solutions such as EPA medium (Buikema et al. 1974; Snell and Persoone 2021), American Society for Testing and Materials (ASTM) reconstituted water (Moreira et al. 2015) or purified water (Ricci 1983; Marotta et al. 2012; Klimek et al. 2013; Olah et al. 2017). While these are often appropriate for the studies mentioned, as they concern freshwater species, these culturing solutions are not necessarily representative of the conditions in a limno-terrestrial environment in which there can be many complex interactions with soils (Xue et al. 2024). This is especially true in the case of interstitial water which contains soil-associated substances such as dissolved salts, colloids and organic matter (de Jonge et al. 2004).

With recent advancements and renewed interest in limno-terrestrial Antarctic microinvertebrates, it is necessary to have established culturing methods to underpin new studies. In this study, we aimed to develop laboratory culturing techniques and conditions based on the interstitial water environment experienced by the indigenous soil-dwelling bdelloid rotifer *Habrotrocha* sp*.* and eutardigrade *A. antarcticus* for use in future laboratory experiments.

## Materials and methods

### Collection of soil and moss samples

Soil samples for elutriate preparation were collected in January 2018 from Robinsons Ridge, East Antarctica. Soils and mosses for the extraction of microinvertebrates were collected from the surrounds of Casey station, East Antarctica (− 66.282S, 110.525E) in January–March 2019 as per McCarthy et al. (2022). The collection procedure was the same for both sampling instances. For the collection of soil samples, the surface was scraped to remove any snow, large stones and debris, the uncovered soil was then loosened to a depth of ~ 5 cm with disinfected (80% v/v ethanol) steel hand shovels and 1–4 kg of soil collected in sterile polypropylene zip-lock bags. Mosses were sampled by carefully removing small (2–3 cm^2^) portions of moss beds with disinfected (80% v/v ethanol) steel spatulas and stored in sterile 50-mL polypropylene centrifuge tubes or sterile polypropylene zip-lock bags. Soils from Robinsons Ridge were used in the preparation of the soil elutriate, while soil and moss samples from Casey station were used to extract rotifer and tardigrade individuals for culturing.

Soil and moss samples for invertebrate extraction underwent no processing and were stored at 4 °C until needed. Soils for elutriate preparation were immediately frozen and upon return to Australia air dried and sieved to ≤ 2 mm and stored at − 20 °C in the dark until needed.

### Microinvertebrate extraction and identification

Upon return to Australia, rotifers and tardigrades were extracted from refrigerated soil and moss samples collected from Casey station, Antarctica, by hand picking as per McCarthy et al. (2022). Extracted organisms were sorted visually by taxa and kept in petri dishes containing ultrapure water and a small amount of detritus from the initial extractions to provide food while culturing procedures were established.

The microinvertebrates used in this experiment were a bdelloid rotifer and a eutardigrade; the microinvertebrates (and algae food source) were identified genetically through Sanger sequencing by EnviroDNA (Enviro Pty. Ltd.) (all rotifer and algae sequences, tardigrade 18S, 28S and ITS2 sequences) and Australian Genome Research Facility (AGRF Pty Ltd.) (tardigrade COI sequences). Each organism was amplified with multiple primers to account for potential amplification failure (Supplementary information Table S1). The rotifer was sequenced using the mitochondrial cytochrome C oxidase subunit I (COI) using four primers to account for amplification failure due to genetic divergence common with these taxa. The tardigrade was sequenced at four loci: nuclear 18S and 28S ribosomal RNA, an internal transcribed spacer between rRNA genes (ITS2) and COI. Sequences were amplified in duplicate and ranged from 76 to 924 bp in length (mean 401 bp) and were matched to EnviroDNA’s in-house database, SILVA (www.arb-silva.de), and Barcode of Life Database (BOLD, www.boldsystems.org) as relevant. Sequences utilised for identification were uploaded to BOLD (see supplementary information Table S1 for sequence database references).

Partial matches were found to various bdelloid genera including *Habrotrocha* (92.03%; family Philodinidae), *Adineta* (91.96%; family Adinetidae) and *Abrochtha* (89.77%; family Philodinavidae); however, deep divergence between these genera and the present sample suggests a not previously sequenced species and prevented confident identification lower than class Bdelloidea. Morphology was therefore used to identify to genus. Using the key by Ricci and Melone (2000), *Habrotrocha* was the only likely match, as *Adineta* lack the prominent trochi of the present species and *Abrochtha* have a distinctive trochi pattern not seen in the present species and a ‘shallow’ mastax, where the present species is ‘deep’. The rotifer was therefore identified as *Habrotrocha* sp. and is referred to as such herein (see supplementary information Table S1 for sequence database references).

Homologous sequences were found to 18S and 28S ribosomal RNA with multiple BOLD entries for *Acutuncus antarcticus* (> 90%), with no close matches to any other organisms. COI sequences had very high (> 99%) matches for *A. antarcticus* in BOLD. Sequence divergence from identified samples of *A. antarcticus*, for the RNA, may be an example of high genetic diversity within the species as described in the literature (Czechowski et al. 2012; Cesari et al. 2016; Tsujimoto et al. 2019). However, close (> 99%) matches for the COI sequences greatly increase the confidence of the identification as *A. antarcticus*; therefore, throughout the text, the tardigrades will be referred to as such (see supplementary information Table S1 for sequence database references).

### Algae food isolation and identification

A culture of the indigenous unicellular green algae *Chlorella* sp. was isolated from Robinson’s Ridge soil samples to provide a food source for cultured microinvertebrates. The algal culture was isolated by streaking a saturated paste of the soil on Bolds Basal Medium (BBM) agar plates (7.5 g L^−1^ agar and 0.705 g L^−1^ commercial Bold’s Basal Medium; PhytoTechnology Laboratories B1675) and incubating them under general test conditions until green colonies were visible to the naked eye. A colony was then sampled and cultured in full-strength liquid BBM in the same conditions as the plates. Once the initial culture had reached a sufficient density (visibly green in solution), it was purified through serial dilution and restarted from a single cell. Purified cultures were kept in half-strength BBM to maintain a stable culture density.

The algae was sequenced at two plastid genes, *tufA* and *rbcL*, and two nuclear loci, rRNA ITS2 and the V4 region of the 18S gene. Homologous sequences were found for 18S, ITS2 with algae in the family Chlorellaceae, specifically for the genera *Chlorella* (89.60%) and *Micractinium* (90.40%). Variation compared to reference sequences suggests a species not previously sequenced. Identification was further refined by morphology to *Chlorella* sp. due to the lack of spines that are indicative of *Micractinium* (Luo et al. 2006). Herein, the algae will be referred to as *Chlorella* sp. (see supplementary information Table S1 for sequence database references).

### Culture media

Two culture media were tested in the study: a soil elutriate prepared by extracting the water-soluble components from an Antarctic soil and balanced salt solution (BSS), a standard artificial solution prepared using laboratory reagents (Piggott et al. 2000). Soil elutriate was chosen to simulate the conditions experienced by the invertebrates in the interstitial water of soil. The second culture media, BSS, was chosen as it is readily prepared from common laboratory reagents and has been shown to be effective in the culturing of soil-dwelling nematodes (Piggott et al. 2000) including the endemic Antarctic nematode *Plectus murrayi* (Brown et al. 2020). Previous studies have cultured aquatic rotifers in various freshwater solutions, but these media were not tested here as the goal was to provide a soil-like media for terrestrial populations of microinvertebrates to improve the environmental relevancy.

To produce the soil elutriate, soils were defrosted, autoclaved (121 °C, 60-min hold) and combined to make a composite sample of 4.5 kg, with ~ 100 g subsamples of each contributing soil, and of the combined soil, reserved for characterisation. A 1:1 ratio (v/v) of soil and ultrapure water was added to borosilicate glass bottles and mixed on an orbital mixer at ~ 32 rpm for 24 h. After mixing, the soil was left to settle for 12 h, transferred to sterile 50-mL polypropylene centrifuge tubes then centrifuged at 3200 rpm for 40 min. The supernatant was then transferred into a fresh tube and centrifuged again at the same settings. After the second centrifuging, the solution was filtered through 0.45-µm nylon syringe filters (FilterBio) into a large beaker (5000 mL) before being transferred into sterile 50-mL centrifuge tubes and stored at − 20 °C. When needed, tubes were defrosted at room temperature and then centrifuged at 3200 rpm for 10 min to remove suspended fine particles flocculated by the freeze–thaw process.

The BSS was prepared by the addition of NaCl 0.417, KCl 0.064, MgCl_2_ 0.328, CaSO_4_·2H_2_O 0.887, Ca(NO_3_)_2_ 0.569 and MgSO_4_·7H_2_O 0.426 g/L to ultrapure water. The pH of BSS was not adjusted as per previous studies (Piggott et al. 2000; Brown et al. 2020) to enable a more direct comparison with soil elutriate pH conditions.

#### Soil and culture media characterisation

Soil samples used for elutriate preparation were characterised to determine their physio-chemical properties. Soil pH and electrical conductivity (EC) were tested on a 1:5 soil/water solution (Rayment and Lyons 2011). Exchangeable cations were measured using inductively coupled plasma mass spectrometry (ICP-MS; Agilent 7900) following 1 M ammonium chloride extraction (Rayment and Lyons 2011). Total organic carbon (TOC) was measured by total organic carbon analyser vaporisation (Skalar Primacs 100). Kjeldahl nitrogen and pseudo-total nutrients (Ca, Mg, P, K, S) were measured by flow injection analysis. For metals and metalloids (Al, Ag, As, B, Ba, Be, Bi, Cd, Co, Cr, Cu, Fe, Hg, Mn, Mo, Na, Ni, Pb, Sb, Se, Sn, Ti, Tl, U, V, Zn), soil was aqua regia digested at 95–105 °C for 60 min followed by ICP-MS analysis (USEPA 1992, 1994, 2014). These measurements were taken for each individual sample and for the combined composite soil which was used for elutriate preparation (recorded measurements of all elements is available in Supplementary information S2). Soil moisture (105 °C for ≥ 8 h) was determined to report soil characteristics on a dry weight basis.

The pH and EC of the culture media were measured with their respective probes of a benchtop multi-channel meter (Meter Toledo SevenExcellence; InLab Expert Pro-ISM (pH) and InLab 731-ISM (EC)).

### General testing conditions

All cultures and trials in this study were kept in an incubator (LabWit ZXSD-R1160) at 10-15°C with a 12/12 light cycle (17-24 μM/m^2^/s Photosynthetically Active Radiation). This is within the upper range of the surface soil temperatures reported during the summer at Casey station, Antarctica (McWatters et al., 2016). All glassware was sequentially detergent washed, rinsed with ultrapure water (>18.2 MΩ m), acid washed in 10% HNO_3_ for at least 24 h, and then rinsed in ultrapure water before drying and use.

### Starter cultures

A starter culture of *Habrotrocha* sp. was prepared in a 35-mm petri dish containing ~ 70% soil elutriate (diluted with ultrapure water) by adding 5 individuals from a culture previously narrowed to a single rotifer individual to ensure a single-species culture. The starter culture was prepared by adding 3.2 mL soil elutriate and 1.0 mL *Chlorella* sp. culture (0.01 g mL^−1^), to 0.4 mL transfer water containing 5 individuals. The culture was then left under general testing conditions and checked weekly to assess population health and growth. After ~ 3 weeks, enough adequately sized individuals were present, and testing commenced. *A. antarcticus* cultures were started in the same way as for the rotifers, except they were slower to reproduce, taking ~ 12 weeks to reach sufficient population size to start testing.

### Comparing the effects of culture media type on population growth

Population growth for *Habrotrocha* sp. and *A. antarcticus* in soil elutriate and BSS was determined. For each species, ~ 100 similarly sized individuals were isolated from their respective starter cultures into isolation dishes containing 70% soil elutriate and 30% ultrapure water. From these isolation dishes, 5 individuals were randomly allocated into each of eight 35-mm petri dishes for each species. Each individual was transferred from the isolation dishes via micropipette with 0.08 mL of the starter culture solution for a total of 0.4 mL of starter culture solution in each dish. A 0.1 mL aliquot of a *Chlorella* sp. culture (0.01 g mL^−1^) was added to all dishes, and then 3.2 mL of either the soil elutriate or BSS for a total of four dishes containing each. Thus, the final volume in each dish was 3.7 mL, with a final media concentration of 86% BSS or soil elutriate.

Prepared dishes were enclosed within larger 200-mm petri dishes to minimise water loss due to evaporation, with locations re-randomised after each observation. Individual dishes were also weighed at day 0 and approximately every 14 days throughout the trials and ultrapure water added as needed to counteract evaporation and ensure consistent media concentration for the duration of the tests.

Rotifer and tardigrade population sizes were assessed using a stereomicroscope (Leica S9i, 6–55 ×), with the total number of active (as per McCarthy et al. (2022)) individuals per dish counted at each observation. Individuals were recorded as active if they were visibly moving, swimming, or feeding. Observations initially occurred at 3- to 4-day intervals and were extended to ~ weekly intervals towards the end of the observation period, for a total duration of 60 days for *Habrotrocha* sp. and 160 days for *A. antarcticus.*

### Assessing the effects of soil elutriate concentration on *Habrotrocha* sp. population growth

*Habrotrocha* sp. population growth was assessed over a series of soil elutriate dilutions (0, 20, 40, 60, 80 and 100%) prepared by diluting the full strength (100%) soil elutriate with ultrapure water. One rotifer from the starter culture was transferred into each well of a sterile polystyrene 24-well plate in 20 µL of starter culture solution. To this, 3.2 mL of each soil elutriate dilution was added to 4 replicate wells in a well plate. This resulted in 4 replicate wells of each of the 6 elutriate dilutions. Additionally, 50 µL of a *Chlorella* sp*.* culture (0.01 g mL^−1^) was added to each well. Thus, final soil elutriate concentrations in the well plates were 0, 20, 39, 59, 78 and 98% in the 0, 20, 40, 60, 80 and 100% soil elutriate treatments respectively.

Wells were observed weekly from day 15 to day 36 under stereomicroscope (Leica Wild M8, 6–40 ×) with the total number of active individuals per well counted at each observation and assessed as per the previous assay. Due to the variable reproductive output and slow reproductive rate of *A. antarcticus* observed in the previous assay, *A. antarcticus* was not able to be investigated.

### Statistical analysis

All statistical analyses were conducted using R 4.0.5 (R Core Team 2019). Differences in the number of active individuals between treatments (growth media type or elutriate concentration) were assessed via 2-factor ANOVA (time × growth media or elutriate concentration) and Tukey’s multiple comparison tests (*p* < 0.05 for statistical significance). Levene’s test was used to confirm homogeneity of variance (*p* > 0.05).

Growth curves were generated for media comparison using the *drc* package (Ritz et al. 2015) by fitting all 25 models within the package to the data. Model fit was determined using Akaike information criterion (AIC) and lack of fit, with a good fitting model having both a low AIC and non-significant lack of fit (*p* > 0.05). The best fitting model in all cases was a Weibulll type 1, 3-parameter model (see supplementary information S3 for model coefficients). All figures were prepared using *gglpot2* and *ggpubr* (Wickham 2016; Kassambara 2020).

## Results

### Culture media properties

The pH and EC of the prepared culture solutions were measured directly. BSS, being a homogenised solution, was measured singly and had a pH of 4.9 and EC of 3145 µS cm^−1^. As the soil elutriate has more potential for variation, being prepared from natural soils, it was characterised by measuring four replicates of the homogenised solution and had a pH of 5.2 ± 0.2 and EC of 417 ± 21 µS cm^−1^. Additionally, as the soil elutriate itself was unable to be analysed due to logistical issues, the soils used in the preparation of the soil elutriate were analysed in their stead (Table 1).

**Table 1:** Characteristics of soil samples from Robinsons Ridge, East Antarctica (select metals reported here, full analysis in Supplementary information S2)

Sample ID	pH	EC	CEC	TOC	N	Cu	Cd	Ni	Pb	Zn
	-	µS cm^−1^	meq 100 g^−1^	%	mg kg^−1^					
169129	5.8	22.7	0.71	< 0.1	280	14	< 0.4	5.1	< 5	21
169131	6.2	30.1	1.60	0.4	790	16	< 0.4	5.5	< 5	28
169133	6.3	22.1	2.00	1.4	2500	29	< 0.4	8.4	< 5	40
169136	6.7	53.1	2.20	0.6	590	19	< 0.4	7.4	< 5	42
169137	6.5	35.7	0.94	< 0.1	280	19	< 0.4	6.4	< 5	39
Composite soil	6.3	33.0	1.10	0.5	820	18	< 0.4	6.7	< 5	33

### Effects of media type on the population growth of *Habrotrocha* sp. and *A. antarcticus*

#### *Habrotrocha* sp.

**Fig. 1:**
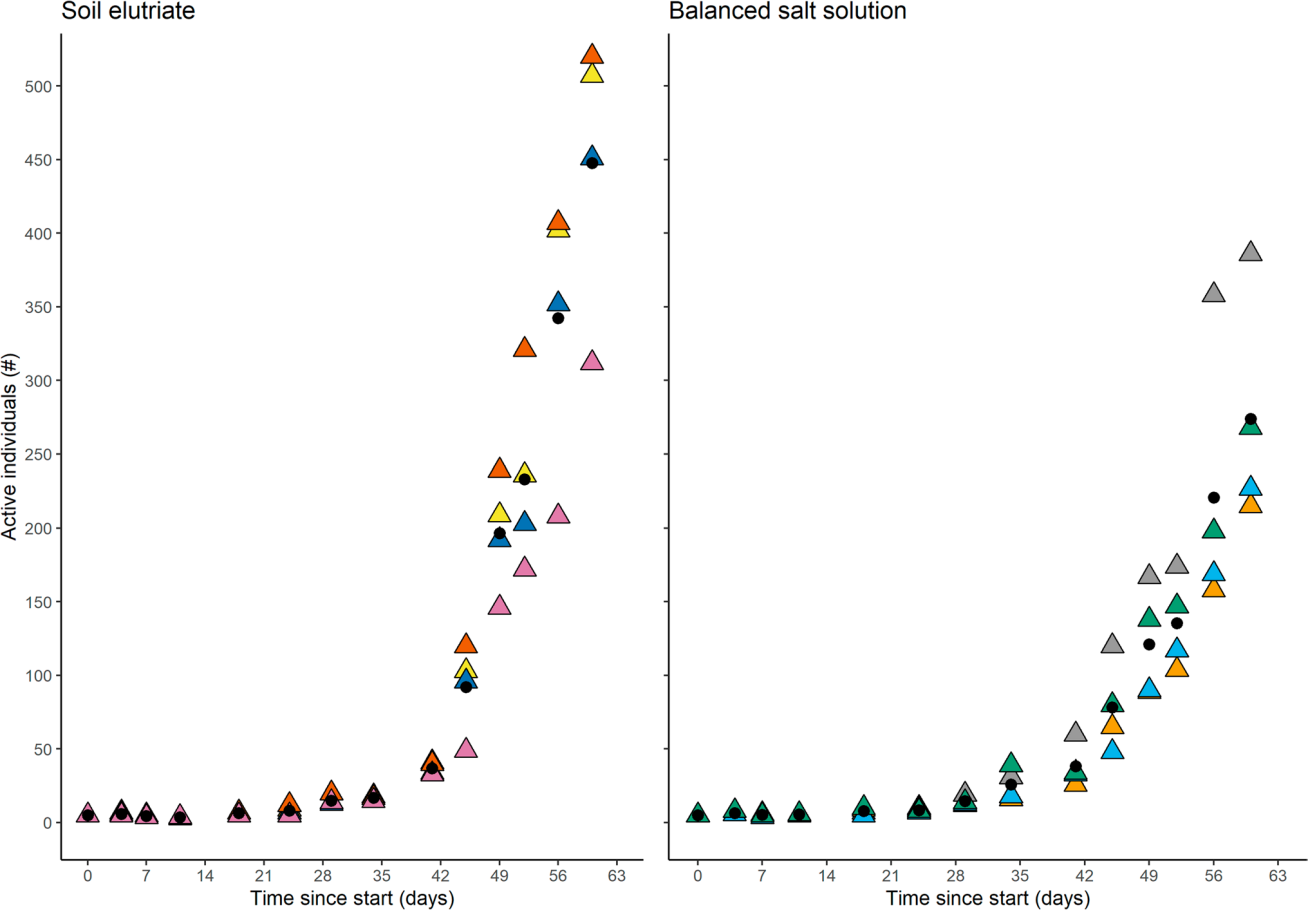
Comparison of population size of the limno-terrestrial Antarctic rotifer *Habrotrocha* sp. cultured in soil elutriate and BSS over the 60-day growth period. Coloured triangles represent the population count of a replicate dish; black points indicate mean of the replicates for each culture media at each observation time

There was no significant difference in rotifer population size between the soil elutriate and the BSS media for the first 52 days (*p* > 0.05, Fig. 1). Significant differences were observed between the media types by day 56 (*p* < 0.01) with significantly larger rotifer populations in the soil elutriate compared to BSS (Supplementary Table S3).

Populations in both culture media tested grew considerably over the 60-day test period (*p* < 0.0001), reaching a mean (± SD) of 448 ± 95 rotifers for the soil elutriate and 274 ± 78 rotifers for BSS (Fig. 1). Population growth between replicates within each culture media varied substantially towards the end of the observation period. The variance was more pronounced in the soil elutriate than in BSS (Fig. 1). While soil elutriate treatments had significantly larger populations at the end of the test period compared to BSS treatments, rotifer populations in both solutions had large population growth of at least 43 × the original population over the 60 days. Thus, both media were suitable for rotifer culturing and reproduction.

Using the fitted Weibull model, the time to reach 50, 100 and 250 individuals was estimated to be 42 (35–48), 45 (35–56) and 52 (32–73) days (mean with 95% CI in parenthesis) in the soil elutriate respectively and 41 (1–82), 48 (0–100) and 59 (0–134) days for BSS as calculated from model coefficients (supplementary information Table S4). Time estimates were similar between the soil elutriate and BSS; however, uncertainty of estimates was higher for rotifer populations growing in the BSS than in the elutriate.

#### *A. antarcticus*

**Fig. 2:**
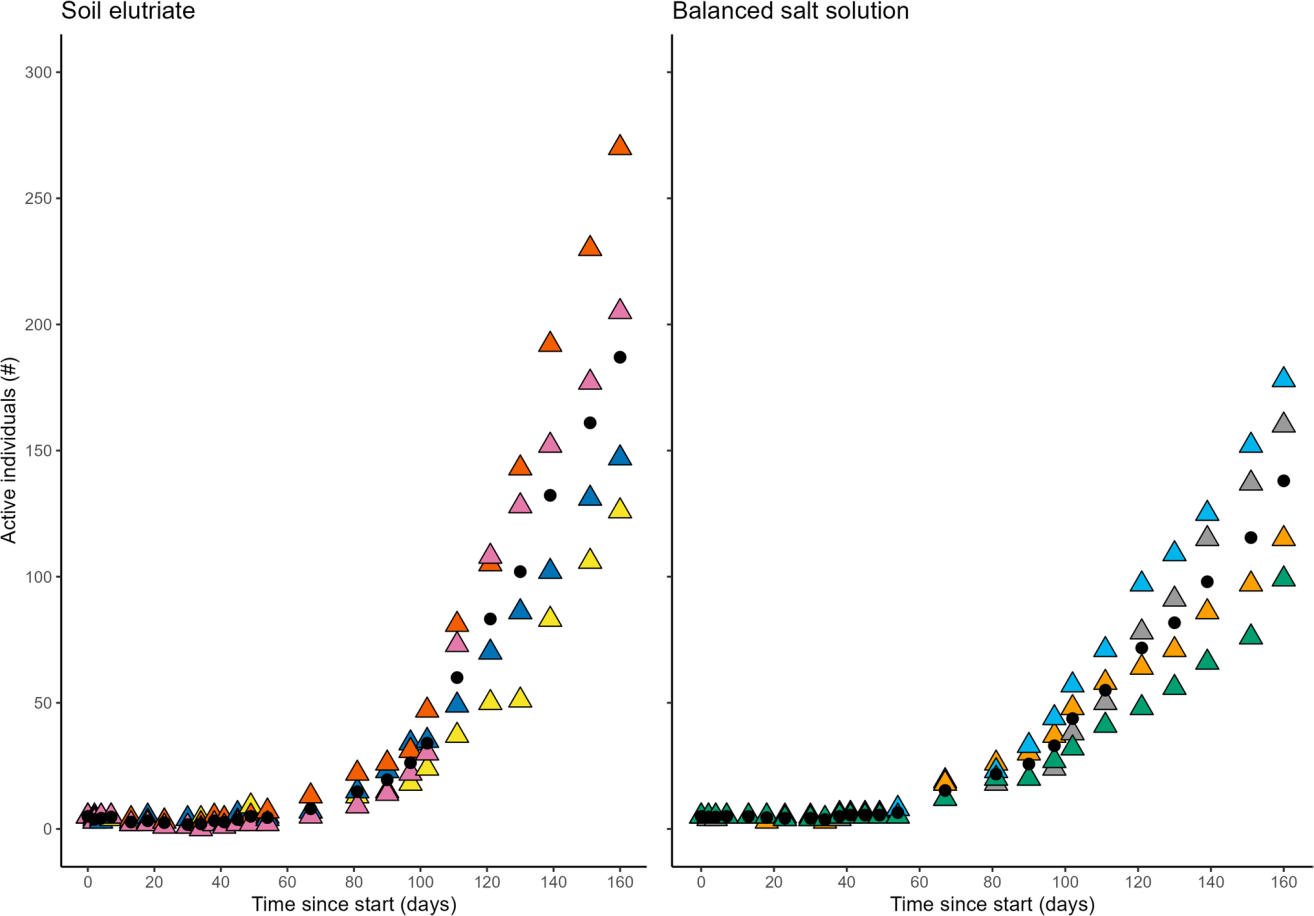
Comparison of population size of the limno-terrestrial Antarctic tardigrade *A. antarcticus* cultured in soil elutriate and BSS over the 160-day growth period. Coloured triangles represent the population count of a replicate dish; black points indicate mean of the replicates for each culture media at each observation time

In contrast to the rotifers, there was no significant difference in population growth of tardigrades in the two media at each time point over the 160-day trial (*p* > 0.05, Fig. 2; Supplementary Table S5). Substantial population increases were observed in both media, reaching a mean (± SD) of 187 ± 65 tardigrades for the soil elutriate and 138 ± 37 tardigrades for BSS at the end of the test (Fig. 2). Tardigrade population growth between replicates within each culture media also varied substantially towards the end of the observation period and to a greater degree than the rotifers. As with the rotifers, the variance was more pronounced in the soil elutriate than in BSS (Fig. 2).

Time estimates were calculated to determine the time required to reach a given population size as with *Habrotrocha* sp. In order to reach populations of 50 and 100 *A. antarcticus*, it was estimated to take 109 (97–120) and 128 (107–149) days respectively in the soil elutriate and 108 (71–145) and 140 (78–201) days in BSS. Estimates to reach defined population sizes remained similar regardless of media (*p* > 0.05). The uncertainty in the estimates was larger in the BSS treatments than the soil elutriate. This is likely to have contributed to the lack of significant difference in time estimates for population growth between the two growth media for the test duration.

### Effect of soil elutriate concentration on *Habrotrocha* sp. population growth

**Fig. 3:**
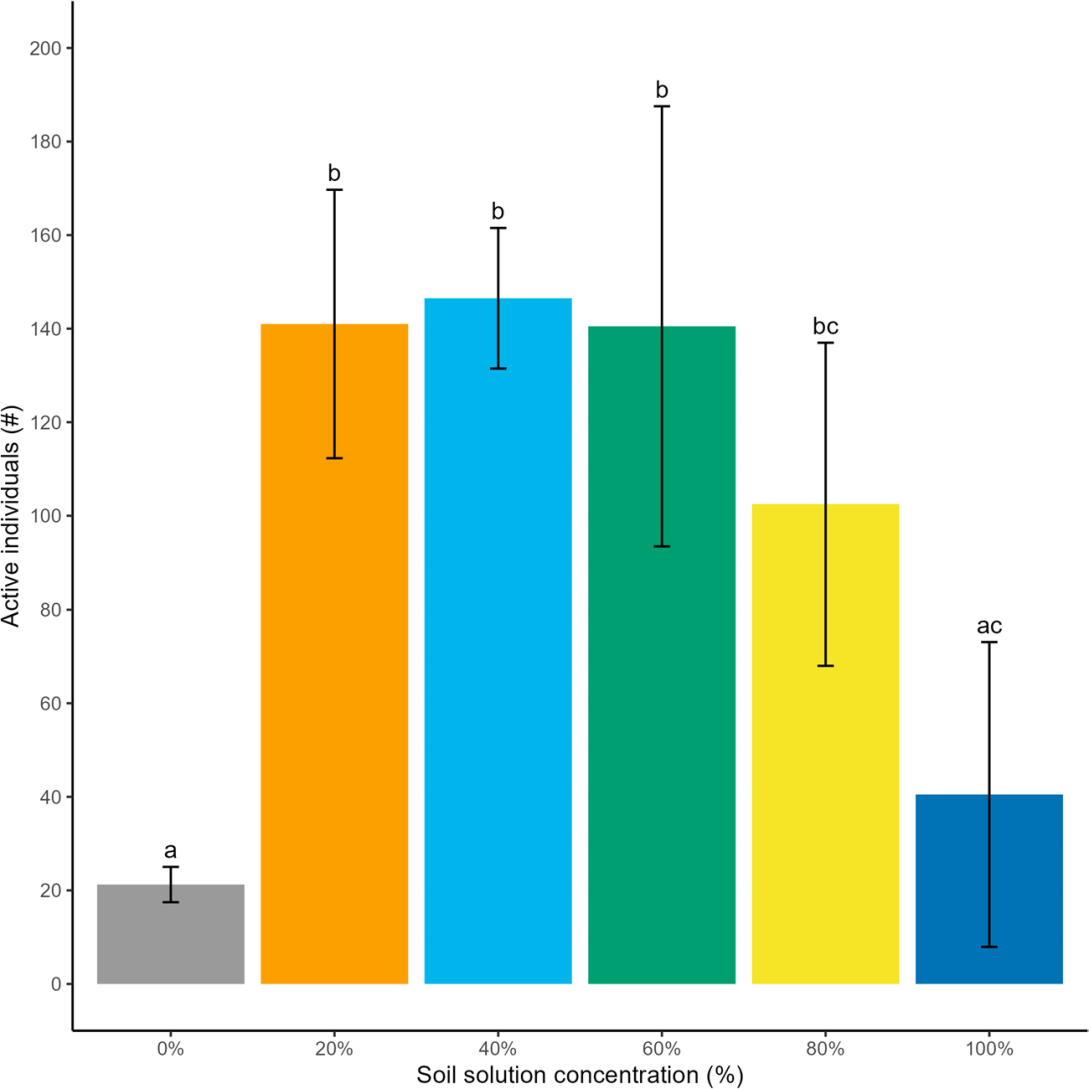
Active rotifer individuals (mean ± SD, *n* = 4) after 36 days at a range of soil elutriate concentrations where columns with different letters above them are significantly different (*p* < 0.05)

Soil elutriate was successful as a culture medium for the population growth of *Habrotrocha* sp.. After 36 days, the 20–80% elutriate treatments had the highest number of active individuals of the concentrations tested with no significant differences in final population size between the treatments (*p* > 0.05) (Fig. 3). In comparison, the 100% elutriate treatments had significantly fewer active individuals than the 20–60% elutriate treatments (*p* < 0.01) but was not significantly different from the 0% or 80% treatments (*p* > 0.05) (Fig. 3). Thus, the 100% elutriate treatment resulted in a culturing environment as inhibitory to population growth over time as ultrapure water (0% elutriate). While there was no statistical significance in population growth between the 20, 40, 60 and 80% dilutions at 36 days, the 40% dilution was determined to be the optimal dilution of soil elutriate for rotifer cultures as this dilution showed the least variation between replicates.

## Discussion

Both the BSS and soil elutriate were suitable for culturing *Habrotrocha* sp. and *A. antarcticus* with substantial population increases occurring during the respective test periods. This is the first time either BSS or a soil elutriate have been utilised for the growth of populations of limno-terrestrial Antarctic rotifers and tardigrades that may also be applicable to non-Antarctic rotifers and tardigrades. We suggest that the choice of media for cultures be selected based on the context of experiment being conducted. In the case of Antarctic or other microinvertebrates from remote locations, BSS is recommended as a routine culturing media to develop and maintain laboratory stocks of both organisms. The artificial nature of the media, its readily available components and ease of preparation make it ideal for routine culture maintenance. In comparison, we suggest using soil elutriates for experiments where site-specific environmental relevance is critical.

To our knowledge, soil elutriates have not been used as a culture media for microinvertebrates, while BSS has only been utilised as media for nematode cultures (Piggott et al. 2000; Brown et al. 2020). Other studies examining population growth have used different media and are therefore difficult to compare to the results found in this study. Media in these previous studies used soil analogues like artificial soil, sand or plaster (e.g. for mites: Huguier et al. (2015) and collembola: Zortea et al. (2017)) or agar plates (nematodes: Sochová et al. (2006)), while standard aquatic rotifer procedures simulate either freshwater or marine waters (ISO 2016a, b).

While population sizes in both media increased substantially over the test periods, variation between replicates was more pronounced in the tardigrades than in the rotifers. The rotifers tested here were from the class Bdelloidea, so they reproduce solely asexually (Ricci 2017). While the specific mode of reproduction of the tardigrade was not observed, *A. antarcticus* have been recorded to be parthenogenic (Altiero et al. 2015; Tsujimoto et al. 2016). Therefore, reproductive mode was unlikely to be a contributing factor to the observed variation in reproductive output between these two taxa. Varied reproductive output appears to be standard in *A. antarcticus* as observed by Altiero et al. (2015), Tsujimoto et al. (2016) and Tsujimoto et al. (2019), proposed to be an adaptive strategy for unpredictable environments. Both the rotifers and tardigrades were collected from an environment in which conditions favourable for growth can be short and unpredictable (Velasco-Castrillón et al. 2014a), requiring specific adaptions for continued survival. It is possible that the variation in reproductive rate as reflected in population growth observed here is an example of one such adaptation, reproductive bet-hedging. Diversified bet hedging is a risk-spreading strategy where variation in reproductive timing protects against unpredictable environments, providing an evolutionary advantage (Philippi and Seger 1989). As observed in other studies, variation in reproductive interval has the potential to partially shield a population from adverse environmental events with the population spread across a range of reproductive and developmental stages at any given time (Crowley et al. 2015; Tarazona et al. 2017). Regardless, the data obtained shows that either medium is suitable to establish substantially sized laboratory cultures of an Antarctic soil-dwelling bdelloid rotifer *Habrotrocha* sp. and tardigrade *A. antarcticus* over known time periods.

It is unclear why the 100% soil elutriate had lower population growth than the intermediate dilutions, and similar population growth to the ultrapure water treatment. It is unlikely to be due to excess salts, as the EC of the soil elutriate was low and similar population growth inhibition was not seen in BSS treatments in the media comparison trials, despite BSS having considerably higher EC. Population growth may have been inhibited by a low-level toxic effect from concentrations of water-soluble metals naturally present in the soils. Metal concentrations in the soil elutriate were not measured; however, concentrations in the source soils (Table 1) were comparable to other non-contaminated sites in East Antarctica (Deprez et al. 1999; Snape et al. 2001; Koppel et al. 2021).

It is possible that the inhibited population growth in the 0% soil elutriate treatment compared to moderate dilutions was due to a nutrient deficiency in ultrapure water. It is unlikely that the nutrient deficiency affected the algae food source, as algal growth was consistent across dilutions with no observed changes in growth pattern, colour, cell shape or size. It is more likely that the ultrapure water was deficient in a mineral necessary for normal development or reproductive fitness of *Habrotrocha* sp. However, there is little evidence in the literature to support this; ultrapure water was successfully used as a media in toxicity tests with the Antarctic bdelloid rotifers, *Philodina* sp. (McCarthy et al. 2022) and *Adineta editae* (Brown et al. 2023). Additionally, low calcium has been found to be beneficial to the reproductive output of the monogonont rotifer *Mytilina brevispina* (Sincock 1976), and phosphorus deficiency in an algae food source was found to have no impact on population growth of the rotifer of *Brachionus plicatilis* (Matsui et al. 2020). The concentration of dissolved ions (herein measured by electrical conductivity) between dilutions of elutriate was likewise unlikely to have substantially contributed to observed differences in population growth. Aquatic rotifers are regularly cultured in ultrapure water (Ricci 1983; Marotta et al. 2012; Klimek et al. 2013; Olah et al. 2017), and limno-terrestrial Antarctic rotifers in particular have been established as being tolerant to wide ranges of ECs in soils (Velasco-Castrillón et al. 2014b), reflecting of their exposure to seasonal melt streams. The results of our study differed than that previously reported for other aquatic and limno-terrestrial species, highlighting the importance of further work to understand the environmental requirements of *Habrotrocha* sp. and limno-terrestrial rotifers more broadly.

## Conclusions

To the authors’ knowledge, this is the first published example of a soil elutriate being used for laboratory culturing of tardigrades or rotifers. Herein, we developed practical methods for the establishment and growth of cultures of rotifers and tardigrades sourced from terrestrial environments in Antarctica using two different media. We have demonstrated that for the species tested, a reagent-based culturing medium (BSS) is suitable for establishing and maintaining laboratory cultures of these organisms and that ultrapure water was suboptimal for population growth of *Habrotrocha* sp. These findings suggest that some established culture mediums suitable for freshwater species (e.g. ultrapure or purified water) may not be suitable for limno-terrestrial populations of rotifers and tardigrades, and that a more nuanced approach is necessary when working with species from these environments. While our study focussed on Antarctic species, it is possible that the procedures utilised here could be successfully adapted for limno-terrestrial populations of tardigrades and rotifers from other climate zones. The foundational work presented here will enable further research into the Antarctic species tested, including understanding their life histories, taxonomic traits and their responses to stressors (environmental and human-induced). 

### Supplementary Information

Below is the link to the electronic supplementary material.

Supplementary file1 (DOCX 52 KB)

## Data Availability

Links to public database records of the genetic sequences are available in the supplementary information attached to this work. Soil analysis and microinvertebrate population growth count data are available from the Australian Antarctic Data Centre: 10.26179/10w9-e172.
