# Supplementary material for: Population growth of two limno-terrestrial Antarctic microinvertebrates in different aqueous soil media
Source: Environ Sci Pollut Res Int. 2024 Apr 27;31(22):33086–97. doi: 10.1007/s11356-024-32905-x (PMC11133119; doi:10.1007/s11356-024-32905-x)
Supplement: Supplementary file 1 — Supplementary file1 (DOCX 52 KB) [file 11356_2024_32905_MOESM1_ESM.docx]

Supplementary information

Table S1. Genetic sequence and amplification information for organisms utilised within the present study. Accession numbers refer to GenBank (https://www.ncbi.nlm.nih.gov/genbank/) reference numbers and can be accessed via embedded hyperlinks.

| **Sequence ID** | **Organism** | **Isolate** | **Accession#** | **Gene** | **Length** | **Primer(s)** |
| --- | --- | --- | --- | --- | --- | --- |
| A3_2_F_B09 | *Chlorella* sp. | 1819C034 | [OP312665](https://www.ncbi.nlm.nih.gov/nuccore/OP312665) | ITS2 | 83 | ITS3 |
| A2_1_F_B06 |  |  | [OP312657](https://www.ncbi.nlm.nih.gov/nuccore/OP312657) | 18s | 225 | DIV4for |
| A2_1_R_B06 |  |  | [OP312658](https://www.ncbi.nlm.nih.gov/nuccore/OP312658) |  | 297 | DIV4rev3 |
| A2_2_F_B07 |  |  | [OP312659](https://www.ncbi.nlm.nih.gov/nuccore/OP312659) |  | 252 | DIV4for |
| A2_2_R_B07 |  |  | [OP312660](https://www.ncbi.nlm.nih.gov/nuccore/OP312660) |  | 294 | DIV4rev3 |
| T2_1b_R_D06 | *A. antarcticus.* | 1819CT04 | [OP312667](https://www.ncbi.nlm.nih.gov/nuccore/OP312667) | 28s | 76 | 28S 7bR |
| T2_2b_R_D07 |  |  | [OP312669](https://www.ncbi.nlm.nih.gov/nuccore/OP312669) |  | 80 |  |
| T1_1b_F_D04 |  |  | [OP312662](https://www.ncbi.nlm.nih.gov/nuccore/OP312662) | 18s | 156 | 18S_Tar_Ff1 |
| T1_2b_F_D05 |  |  | [OP312664](https://www.ncbi.nlm.nih.gov/nuccore/OP312664) |  | 150 |  |
| 189400 |  | C036B | [PP316651](https://www.ncbi.nlm.nih.gov/nuccore/PP316651) | COI | 652 | LCOI-1490 (forward)  HCOI-2198 (reverse) |
| 189401 |  | C036B | [PP316652](https://www.ncbi.nlm.nih.gov/nuccore/PP316652) |  | 652 |  |
| 189402 |  | C036B | [PP316653](https://www.ncbi.nlm.nih.gov/nuccore/PP316653) |  | 652 |  |
| 189403 |  | C036B | [PP316654](https://www.ncbi.nlm.nih.gov/nuccore/PP316654) |  | 652 |  |
| B12 | *Habrotrocha* sp. | 1819CT01 | [OQ445492](https://www.ncbi.nlm.nih.gov/nuccore/OQ445492) | COI | 646 | HCO2198 |
| C01 |  |  | [OQ445496](https://www.ncbi.nlm.nih.gov/nuccore/OQ445496) |  | 644 |  |
| C02 |  |  | [OQ445498](https://www.ncbi.nlm.nih.gov/nuccore/OQ445498) |  | 644 | ZplankF1 |
| C03 |  |  | [OQ445499](https://www.ncbi.nlm.nih.gov/nuccore/OQ445499) |  | 644 |  |
| C04 |  |  | [OQ445497](https://www.ncbi.nlm.nih.gov/nuccore/OQ445497) |  | 644 | 30F |
| C05 |  |  | [OQ445493](https://www.ncbi.nlm.nih.gov/nuccore/OQ445493) |  | 644 |  |
| C06 |  |  | [OQ445494](https://www.ncbi.nlm.nih.gov/nuccore/OQ445494) |  | 646 | dgLCO |
| C07 |  |  | [OQ445495](https://www.ncbi.nlm.nih.gov/nuccore/OQ445495) |  | 630 |  |

Table S2. ICP-MS results for analysis of soils used in elutriate preparation. LOR = Limit of reporting for each element

| Element | LOR | Sample | | | | | |
| --- | --- | --- | --- | --- | --- | --- | --- |
| - | - | 169129 | 169131 | 169133 | 169136 | 169137 | Combined |
| - | mg kg^-1^ | | | | | | |
| *Al* | 20 | 4400 | 4900 | 6000 | 6700 | 5700 | 5600 |
| *Ag* | 2 | < 2 | < 2 | < 2 | < 2 | < 2 | < 2 |
| *As* | 2 | < 2 | < 2 | < 2 | < 2 | < 2 | < 2 |
| *B* | 10 | < 10 | < 10 | < 10 | < 10 | < 10 | < 10 |
| *Ba* | 10 | 63 | 80 | 91 | 150 | 100 | 100 |
| *Be* | 2 | < 2 | < 2 | < 2 | < 2 | < 2 | < 2 |
| *Bi* | 10 | < 10 | < 10 | < 10 | < 10 | < 10 | < 10 |
| *Ca* | 5 | 2800 | 3200 | 5700 | 5400 | 6100 | 4400 |
| *Cd* | 0.4 | < 0.4 | < 0.4 | < 0.4 | < 0.4 | < 0.4 | < 0.4 |
| *Co* | 5 | < 5 | < 5 | 12 | 9.8 | 7.9 | 7.6 |
| *Cr* | 5 | 18 | 20 | 15 | 13 | 16 | 18 |
| *Cu* | 5 | 14 | 16 | 29 | 19 | 19 | 18 |
| *Fe* | 20 | 23000 | 28000 | 34000 | 40000 | 34000 | 32000 |
| *Hg* | 0.1 | < 0.1 | < 0.1 | < 0.1 | < 0.1 | < 0.1 | < 0.1 |
| *K* | 5 | 2300 | 3000 | 3900 | 5700 | 3900 | 4100 |
| *Mg* | 5 | 2000 | 2300 | 3600 | 4200 | 3000 | 3200 |
| *Mn* | 5 | 100 | 110 | 140 | 140 | 130 | 120 |
| *Mo* | 5 | < 5 | < 5 | < 5 | < 5 | < 5 | < 5 |
| *Na* | 5 | 190 | 240 | 290 | 240 | 370 | 260 |
| *Ni* | 5 | 5 | 5.5 | 8.4 | 7.4 | 6.4 | 6.7 |
| *P* | 5 | 2200 | 2500 | 2700 | 2700 | 3800 | 3000 |
| *Pb* | 5 | < 5 | < 5 | < 5 | < 5 | < 5 | < 5 |
| *S* | 5 | 67 | 110 | 440 | 200 | 110 | 160 |
| *Sb* | 10 | < 10 | < 10 | < 10 | < 10 | < 10 | < 10 |
| *Se* | 2 | < 2 | < 2 | < 2 | < 2 | < 2 | < 2 |
| *Sn* | 10 | < 10 | < 10 | < 10 | < 10 | < 10 | < 10 |
| *Ti* | 10 | 760 | 890 | 1200 | 1700 | 1100 | 1200 |
| *Tl* | 10 | < 10 | < 10 | < 10 | < 10 | < 10 | < 10 |
| *U* | 10 | < 10 | < 10 | < 10 | < 10 | < 10 | < 10 |
| *V* | 10 | 71 | 84 | 120 | 140 | 110 | 110 |
| *Zn* | 5 | 20 | 28 | 40 | 42 | 39 | 33 |

Table S3 Comparison of population size (mean and standard deviation (SD)) of the limno-terrestrial Antarctic rotifer *Habrotrocha* sp. cultured in soil elutriate balanced salt solution (BSS) over the 60-day growth period. Starting population size of 5 individuals per Petri dish (n=4)

|  | ***n* rotifers** | | | | ***p* value** |
| --- | --- | --- | --- | --- | --- |
| **Time** | **Elutriate** | | **BSS** | |  |
| **days** | **Mean** | **SD^a^** | **Mean** | **SD^a^** |  |
| *4* | 6.0 | 0.8 | 6.5 | 1.0 | 1.0000 |
| *7* | 4.5 | 0.6 | 5.3 | 1.0 | 1.0000 |
| *11* | 3.5 | 0.6 | 5.5 | 0.6 | 1.0000 |
| *18* | 6.5 | 1.0 | 7.8 | 2.2 | 1.0000 |
| *24* | 8.3 | 3.0 | 8.5 | 1.3 | 1.0000 |
| *29* | 15.0 | 3.4 | 14.5 | 3.1 | 1.0000 |
| *34* | 17.0 | 1.4 | 26.0 | 10.9 | 1.0000 |
| *41* | 37.0 | 4.1 | 38.3 | 14.9 | 1.0000 |
| *45* | 92.0 | 30.4 | 78.3 | 30.8 | 1.0000 |
| *49* | 196.5 | 38.9 | 121.0 | 38.3 | 0.5726 |
| *52* | 233.0 | 64.2 | 135.5 | 31.4 | 0.1195 |
| *56* | 342.3 | 92.9 | 220.8 | 93.0 | 0.0087 |
| *60* | 447.5 | 95.2 | 274.0 | 78.0 | <0.0001 |

^a^ standard deviation.

Table S4. Coefficients for modelled population growth for rotifers and tardigrades cultured in balanced salt solution (BSS) and soil elutriate. All models were Weibull type 1, 3 parameter.

|  |  | **Coefficient** | | |
| --- | --- | --- | --- | --- |
| **Organism** | **Culture media** | ***b*** | ***d*** | ***e*** |
| *Habrotrocha* sp. | BSS | -0.82320 | 34445.7 | 405.843 |
|  | Soil elutriate | -2.77821 | 1525.71 | 64.6449 |
| *A. antarcticus.* | BSS | -0.47548 | 16418.7 | 4326.19 |
|  | Soil elutriate | -1.57343 | 860.089 | 209.023 |

Table S5. Comparison of mean population size of the terrestrial Antarctic tardigrade *A. antarcticus* in soil elutriate and balanced salt solution (BSS) over the 160-day growth period. Starting population size of 5 individuals per Petri dish (n=4)

|  | ***n* tardigrades** | | | | ***p* value** |
| --- | --- | --- | --- | --- | --- |
| **Time** | **Elutriate** | | **BSS** | |  |
| **(days)** | **Mean** | **SD^a^** | **Mean** | **SD^a^** |  |
| *2* | 3.8 | 1.0 | 4.8 | 0.5 | 1.000 |
| *4* | 4.3 | 1.0 | 4.8 | 0.5 | 1.000 |
| *7* | 4.8 | 0.5 | 5.0 | 0.0 | 1.000 |
| *13* | 2.8 | 1.0 | 5.0 | 0.0 | 1.000 |
| *18* | 3.3 | 1.3 | 4.5 | 1.0 | 1.000 |
| *23* | 2.5 | 1.0 | 4.3 | 0.5 | 1.000 |
| *30* | 1.8 | 1.5 | 4.3 | 0.5 | 1.000 |
| *34* | 2.0 | 1.6 | 3.8 | 0.5 | 1.000 |
| *38* | 3.3 | 1.5 | 5.0 | 0.8 | 1.000 |
| *41* | 2.8 | 1.5 | 5.5 | 0.6 | 1.000 |
| *45* | 3.8 | 1.7 | 5.5 | 0.6 | 1.000 |
| *49* | 5.0 | 2.9 | 5.5 | 0.6 | 1.000 |
| *54* | 4.5 | 2.1 | 6.5 | 1.7 | 1.000 |
| *67* | 8.0 | 3.5 | 15.3 | 3.8 | 1.000 |
| *81* | 14.8 | 5.4 | 21.8 | 3.5 | 1.000 |
| *90* | 19.5 | 5.9 | 25.8 | 6.8 | 1.000 |
| *97* | 26.3 | 7.5 | 33.0 | 9.2 | 1.000 |
| *102* | 34.0 | 9.8 | 43.8 | 11.0 | 1.000 |
| *111* | 60.0 | 20.5 | 55.0 | 12.7 | 1.000 |
| *121* | 83.3 | 28.1 | 71.8 | 20.8 | 1.000 |
| *130* | 102.0 | 41.7 | 81.8 | 23.1 | 0.9974 |
| *139* | 132.3 | 49.3 | 98.0 | 27.0 | 0.9480 |
| *151* | 161.0 | 54.6 | 115.5 | 35.1 | 0.8123 |
| *160* | 187.0 | 64.6 | 138.0 | 37.1 | 0.7517 |

^a^ standard deviation.
